# The Physicochemical Properties and Antioxidant Activity of Spirulina (*Artrhospira platensis*) Chlorophylls Microencapsulated in Different Ratios of Gum Arabic and Whey Protein Isolate

**DOI:** 10.3390/foods11121809

**Published:** 2022-06-20

**Authors:** Zhi-Hong Zhang, Bangjie Yu, Qili Xu, Zhenyu Bai, Kai Ji, Xianli Gao, Bo Wang, Rana Muhammad Aadil, Haile Ma, Rensong Xiao

**Affiliations:** 1 Key Laboratory of Modern Agricultural Equipment and Technology (Jiangsu University), Ministry of Education, School of Food and Biological Engineering, Jiangsu University, Zhenjiang 212013, China; zhihong1942@ujs.edu.cn (Z.-H.Z.); 15658085150@163.com (B.Y.); yybangki@163.com (Q.X.); bzy18305110782@163.com (Z.B.); jk18852855865@163.com (K.J.); gaoxianli@ujs.edu.cn (X.G.); wangbo670@163.com (B.W.); 2 National Institute of Food Science and Technology, University of Agriculture, Faisalabad 38000, Pakistan; muhammad.aadil@uaf.edu.pk; 3 Suqian Huiwei Food Co., Ltd., Suqian 223900, China; klcszl@163.com

**Keywords:** spirulina, microencapsulation, freeze-drying, physicochemical properties, antioxidant activity

## Abstract

Spirulina (*Artrhospira platensis*) is rich in chlorophylls (CH) and is used as a potential natural additive in the food industry. In this study, the CH content was extracted from spirulina powder after ultrasound treatment. Microcapsules were then prepared at different ratios of gum Arabic (GA) and whey protein isolate (WPI) through freeze-drying to improve the chemical stability of CH. As a result, a* and C* values of the microcapsules prepared from GA:WPI ratios (3:7) were −8.94 ± 0.05 and 15.44 ± 0.08, respectively. The GA fraction increased from 1 to 9, and encapsulation efficiency (EE) of microcapsules also increased by 9.62%. Moreover, the absorption peaks of CH at 2927 and 1626 cm^−1^ in microcapsules emerged as a redshift detected by FT-IR. From SEM images, the morphology of microcapsules changed from broken glassy to irregular porous flake-like structures when the GA ratio increased. In addition, the coated microcapsules (GA:WPI = 3:7) showed the highest DPPH free radical scavenging activity (SA_DPPH_) (56.38 ± 0.19) due to low moisture content and better chemical stability through thermogravimetric analysis (TGA). Conclusively, GA and WPI coacervates as the wall material may improve the stability of CH extracted from spirulina.

## 1. Introduction

Spirulina (*Artrhospira platensis*) belongs to the phylum *Cyanobacteria*, the family of *Oscillatoriaceae* that are known as blue-green algae [1]; it also contains proteins (phycocyanin), B-group vitamins, natural pigments (chlorophyll and carotenoids) and essential fatty acids. Spirulina have been widely recognized as a food and dietary supplement since the 16th century [2,3]. Recent research studies prove that spirulina has numerous health benefits such as antioxidant [4], immunomodulatory [5] and anti-inflammatory activities [6]. However, it has an undesirable odor and flavor, which may cause anaphylaxis in some people, especially in young people, which impedes its application in the food industry [7,8]. Therefore, an effective strategy is required to expand its application through the extraction of the non-allergenic biologically active compounds from spirulina, such as C-phycocyanin, carotenoids and lipids, etc. [3,9,10].

Chlorophylls (CH) are widely distributed in higher plant leaves and fruits and present a brilliant and cheerful green color, as well as numerous bioactivities in various in vivo and in vitro testing, which could be used as potential natural green pigments to replace the synthetic colorants in the food industry [11,12]. In spirulina, CH is the major photosynthetic greenish pigment. Its content of CH a is from 0.8% to 1.5% of dry weight, which is higher than the total CH (sum CH a and b) in other species, such as cassava leaves (326.27–747.89 mg/100 g dry weight), chicory (383.79 mg/100 g dry weight), dandelion (278.52 mg/100 g dry weight) and rocket (439.86 mg/100 g dry weight) [13,14,15]. Therefore, spirulina can be considered an important extraction resource for CH. However, CH is unstable and can be easily degraded by environmental conditions, such as temperature, pH value, light intensity and enzymes, resulting in their color change from green to colorless, and thereby losing their application value and health benefits [16,17,18]. Moreover, CH is practically insoluble in water, which also limits its application in the food industry.

Recently, microencapsulation technology has attracted attention in the food industry [19,20]. For this technology, the stability and water solubility compounds (wall matrix) are used alone or in combination to encapsulate the sensitive compounds (core matrix) [21,22]. Proteins (whey protein, soybean protein, gelatins, etc.), polysaccharides (gums, maltodextrins, modified starches and chitosan, etc.) and lipids (phospholipids) are generally selected as common wall materials for encapsulating active compounds [19]. Among these biopolymers, gum Arabic (GA) is characterized by low viscosity, high water solubility and emulsifying properties, while whey protein isolate (WPI) has the better coating ability of hydrophobic compounds and nutritional benefits, which are the most popular wall materials with encapsulated hydrophobic active compounds [23,24,25].

However, there are no studies with detailed investigations on the physicochemical and antioxidant properties of CH derived from spirulina using the co-carrier agents of GA and WPI by freeze-drying method. Therefore, the analyses for physicochemical properties of microcapsules with different ratios of GA:WPI were carried out by chromameter, FT-IR, TGA and SEM; antioxidant activity was determined by DPPH free radical scavenging activity to evaluate the protection ability against CH with the co-carrier in this study.

## 2. Material and Methods

### 2.1. Materials

Commercially dried spirulina (*A. platensis*) powder was purchased from Lijiang Shengbo Biological Engineering Co., Ltd. (Lijiang, China). The biomass was packed in an aluminum foil bag and stored at 4 °C. Absolute ethanol of analytical grade was purchased from Sinopharm Chemical Reagent Co., Ltd. (Shanghai, China). GA powder of biochemical reagent was purchased from Shanghai Aladdin Biochemical Technology Co., Ltd. (Shanghai, China). WPI (90% protein, 2.5% carbohydrates, 0.6% fats and 0.5% salt) was purchased from Myprotein Co., Ltd. (Cheshire, UK). 1,1-Diphenyl-2-picrylhydrazyl (DPPH) was purchased from Sigma-Aldrich, Inc. (St. Louis, MO, USA).

### 2.2. Extraction CH from Spirulina

10 g of dried spirulina powder was blended with 100 mL of absolute ethanol at room temperature. The particles were stirred until they were completely soaked. The CH extraction was used by an ultrasonic cell crusher instrument (SCIENTZ-IID, Ningbo Scientz Biotechnology Co., Ltd., Ningbo, China) at 350 rpm in a magnetic stirrer (MS-PA, Xi’an Endian Biotechnology Co., Ltd. Xi’an, China). The ultrasound system used had the power of 350 W at 20–25 kHz frequency for 5 min (5 s on and 5 s off) at room temperature. After the extraction, the mixture was separated by a centrifuge (DL-6-B, Shanghai Anting Scientific Instrument Factory, Shanghai, China) at 4000× *g* for 10 min. For sufficient extraction of CH, the extraction procedures were repeated 5 to 7 times. Subsequently, the collected supernatant was concentrated by a rotary vacuum evaporator at 40 °C for 10 min. Finally, the concentrated CH solution was fixed to a 500 mL volumetric flask with absolute ethanol and stored at 4 °C to prepare the CH-microcapsules.

### 2.3. Preparation of Microcapsules by Freeze-Drying

GA and WPI powder (wall matrix) were dissolved in deionized water (2%, *w*/*v*) while stirring at 40 °C for 1 h. Subsequently, the polymer solutions were cooled to room temperature and kept overnight in a refrigerator to ensure complete hydration. Then, 30 mL of concentrated CH solution was blended with the different volume ratios of the GA and WPI solution according to the volume ratio of 1:2 using a magnetic stirrer at 800 rpm. The blending procedure of the core matrix and wall matrix solution is shown in Table 1. Finally, the mixed solution was pre-frozen at −80 °C for 6 h and then dried with a vacuum freeze dryer (SCIENTZ-10 N, Ningbo Scientz Biotechnology Co., Ltd., Ningbo, China) for 48 h. The prepared microcapsules were collected into an aluminum foil bag and kept in a desiccator to avoid degradation.

### 2.4. Determination of the Visual Color of Microcapsule

The visual color of each microcapsule was measured by a chromameter (CR-400, KONICA MINOLTA, Inc., Tokyo, Japan) at room temperature, as described in a previous study [26]. The measured data were expressed as the Hunter color values, i.e., L*, a* and b* value, which represents the lightness (0 to 100), greenness (-) to redness (+) and yellowness (+) to blueness (-), respectively. Moreover, the chroma (C*) was calculated by Equation (1), which indicated the color intensity of samples [27]:

(1) $$C^{*}=\sqrt{a^{*2}+b^{*2}}$$

### 2.5. Encapsulation Efficiency (EE) of CH

The EE of CH was determined according to the method reported by Zhang et al. [25] with some modifications. A precise weight of 50 mg of each microcapsule was added to 5 mL of absolute ethanol while stirring for 10 min, and then the mixture was centrifuged at 9000× *g* for 10 min to obtain the supernatant A. Another sample (50 mg) was added to 5 mL of ethanol with ultrasound treatment at 350 W for 10 min using an ultrasonic cell crusher instrument (SCIENTZ-IID, Ningbo Scientz Biotechnology Co., Ltd., Ningbo, China). Then, the supernatant B was obtained by centrifugation at 9000× *g* for 10 min. The CH contents of supernatant A and B representing the surface chlorophylls (S_CHs_) and total chlorophylls (T_CHs_) of microencapsulation samples, respectively, were determined through a UV-Vis spectrophotometer (UV-1601, Beijing Beifen-Ruili analytical instrument Co., Ltd., Beijing, China) and calculated by Equation (2). In this study, the concentration of CH extracted from spirulina was 120.72 μg/mL. Finally, the EE of CH was calculated by Equation (3):

(2) $$C_{Chlorophyll}=6.10\times A_{665}+20.04\times A_{649}$$

where *C_chlorophyll_* represents the concentration of CH (μg/mL); *A*_665_ and *A*_649_ represent the absorbance at 665 and 649 nm, respectively; 6.10 and 20.04 are the transfer coefficients.

(3) $$EE\%=\frac{T_{CHs}-S_{CHs}}{T_{CHs}}\times 100$$

### 2.6. Measurement of Structural Characteristics

The functional groups of CH, GA, WPI and microcapsules were identified from corresponding spectra acquired by Fourier transform infrared spectroscopy (FT-IR) using a NicoletiS50 FT-IR spectrophotometer (Thermo Fisher Scientific Inc., Waltham, MA, USA). The sample was blended with KBr at a mass ratio of 1:50 (*w*/*w*) and pressed into a pellet. The pellet was scanned in the spectral range of 400 to 4000 cm^−1^ with a spectral resolution of 4 cm^−1^ at room temperature. The spectrum of the sample was obtained by automatically removing the background spectrum under identical operational conditions.

### 2.7. Measurement of Microcapsules Morphology

The morphology of the microcapsule was identified using scanning electron microscopy (Quanta 250FEG, FEI Co., Hillsboro, OR, USA). The sample was sputtered with a thin gold layer for 15 s. The sample was scanned at an accelerating beam voltage of 20 kV. The representative image of the sample was obtained at a magnification of ×700.

### 2.8. Determination of UV Light Stability

The UV light stability of the microcapsule was measured using a UV crosslinker (Scientz03-II, Ningbo Scientz Biotechnology Co., Ltd., Ningbo, China) with 312 nm of wavelength following a previous study [28] with some modifications. Briefly, 50 mg of microcapsule was blended with 5 mL of absolute ethanol while stirring. Then, the blended solution was placed in the UV crosslinker for different treatment times (0 to 60 min), and the CH content of the solution was determined at 10 min intervals. Finally, the CH retention rate (RT_CHs_) was calculated by Equation (4):

(4) $$RT_{CHs}=\frac{C_{t}}{C_{0}}\times 100,$$

where *C*_0_ is the original *CH* content of the solution without UV treatment; *C*_t_ is the *CH* content of the solution after t min by UV light treatment.

### 2.9. Determination of Thermal Behaviour

The thermal behaviors of GA, WPI and microcapsules were determined by thermogravimetric analysis (TGA) using a thermogravimetric analyzer (STA449C, NETZSCH Co., Selb, Germany) according to a previous study [29]. 2.0 mg of microcapsule sample was sealed in a standard aluminum (Al_2_O_3_) pan and heated from 25 to 400 °C at a heating rate of 10 °C/min under nitrogen as a protection atmosphere at a flow rate of 20 mL/min. An empty sealed aluminum pan was used as a reference.

### 2.10. Determination of Antioxidant Activity

The antioxidant activity of microcapsules was determined by DPPH free radical scavenging activity using the method described by our previous study [30] with some modifications. A 100 mg microcapsule was blended with 10 mL of deionized water with ultrasonic for 1 min (600 W) at room temperature and then centrifuged at 9000× *g* for 10 min. Subsequently, 3 mL of the supernatant solution was mixed with 2 mL of DPPH solution (0.1 mmol/L, dissolved in absolute ethanol), and then incubated in darkness at room temperature for 30 min. Finally, the absorbance of the sample/DPPH mixed solution was measured at 517 nm by a UV-Vis spectrophotometer. The DPPH free radical scavenging activity (SA_DPPH_) was calculated by the following Equation (5):

(5) $$SA_{DPPH}=(1-\frac{A_{\mathrm{sample}}-A_{\mathrm{blank}}}{A_{\mathrm{control}}})\times 100,$$

where *A*_sample_ is the absorbance of the sample solution; *A*_control_ is the absorbance of the control in which the deionized water replaces the sample solution; *A*_blank_ is the absorbance of the blank in which the DPPH solution was replaced with absolute ethanol.

### 2.11. Statistical Analysis

All experiments were replicated at least three times for each test, and the results were reported as the mean ± standard deviation using SPSS software (version 22.0, IBM Co., New York, NY, USA). Variance analysis was analyzed by Origin software (Version 9.0, Origin Lab, Northampton, MA, USA). Significance analysis was performed by Tukey’s multiple comparison test at 0.05 significance level.

## 3. Results and Discussion

### 3.1. The Visual Color of Microcapsules

The effect of different ratios of GA and WPI as wall material on the visual color of microcapsules is shown in Table 2. The L* values of microcapsules were significantly influenced by the ratios of GA and WPI (*p* < 0.05), which showed a tendency to first decrease and then increase. The lowest value of L* values was obtained for the GA:WPI (5:5), which could be attributed to the difference in the original color of GA and WPI, and the difference in the light refraction of the mixture. This phenomenon was also reported in previous studies, which presented that the lighter wall material and its content can change the L* value of microcapsules [27,31,32]. As for the a* value, all the microcapsules showed negative values, which indicated that the samples presented the greenness, and the microcapsule prepared by GA:WPI ratio in 3:7 showed more greenness than other samples (*p* < 0.05). The lowest b* value was observed for microcapsules prepared for GA:WPI (1:9), which suggested that a higher content of WPI gave the microcapsules more yellowness because of the WPI presenting the light yellowness (*p* < 0.05). The highest C* value was observed for microcapsules prepared with GA:WPI (3:7), which indicated more greenness than other microcapsules covered with GA:WPI (*p* < 0.05). These results indicated that the appropriate increase in the proportion of GA in composite wall material can preserve the greener color of microcapsules, which was consistent with previous studies [27,33].

### 3.2. Encapsulation Efficiency (EE) of CH

The total chlorophylls (T_CHs_), surface chlorophylls (S_CHs_) and EE of microcapsules prepared by different ratios of GA:WPI is shown in Table 3. The T_CHs_ of microcapsules prepared by different ratios of GA:WPI were about 143 μg but did not observe any significant difference (*p* > 0.05). With the increase in GA content, the S_CHs_ of microcapsules gradually decreased. The microcapsules prepared by GA:WPI (9:1) showed the lowest of S_CHs_, (19.38 ± 0.23 μg) followed by those prepared by GA:WPI (7:3 and 5:5), but these samples did not observe any significant difference (*p* > 0.05). As for the EE of microcapsules, it showed an increasing trend when the GA fraction was increased. These results indicate that the higher fraction of GA content in wall material can retain a higher CH content in the microcapsules. This phenomenon could be attributed to the GA with good emulsifying ability in a solution that could form a surface binding ability at the oil-water interface. Although there is a large number of hydrophobic amino acid residues in WPI, their residues are generally distributed in the internal spatial structure under natural conditions, and it is difficult to bind with hydrophobic compounds. These results were consistent with previous studies that showed an optimal EE of core materials covered by GA as wall material [27,34].

### 3.3. Structural Characteristics of Microcapsules

**Figure 1:**
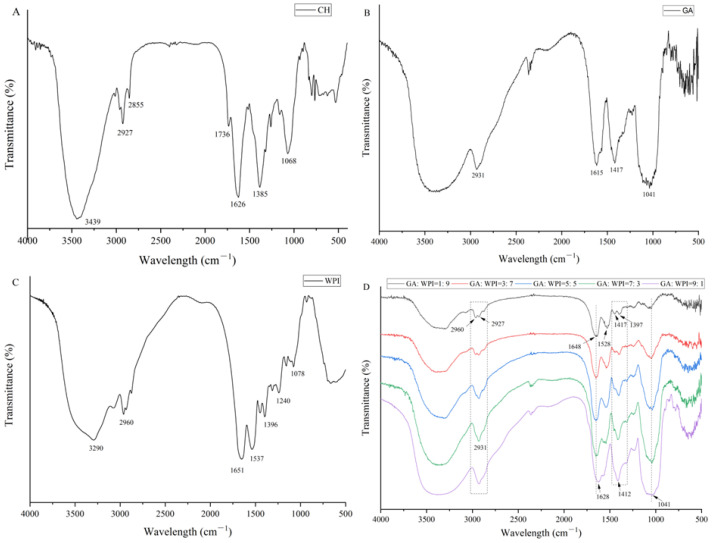
FT-IR spectra of CH (**A**), GA (**B**), WPI (**C**) and microcapsules (**D**) were prepared by different ratios of GA:WPI.

FT-IR spectra of CH, GA, WPI and microcapsules prepared by different ratios of GA:WPI is shown in Figure 1. The absorption peaks of CH (Figure 1A) at 3439 cm^−1^, 2927 cm^−1^, 2855 cm^−1^, 1736 cm^−1^, 1626 cm^−1^, 1385 cm^−1^ and 1068 cm^−1^ represented the functional groups O-H stretching, C-H stretching (phytol), C-H bending (methyl and methylene), C-N stretching, C-O stretching (C-17^3^ and C-13^3^), C-N stretching (aromatic system)/N-H bending and C-O stretching, respectively, which was relevant to previous studies [27,35]. The characteristic absorption peak of GA (Figure 1B), the absorption peaks at 3410 cm^−1^, 2931 cm^−1^, 1615 cm^−1^, 1417 cm^−1^ and 1041 cm^−1^ were associated with O-H stretching, CH- stretching, C=O stretching/N-H bending, C-N stretching and C-O stretching, respectively, which was consistent with previous studies [27,36]. The WPI spectrum (Figure 1C) showed mainly absorption bands at 3290 cm^−1^, 2960 cm^−1^, 1652 cm^−1^, 1537 cm^−1^, 1396 cm^−1^, 1240 cm^−1^ and 1078 cm^−1^, which were indicated O-H stretching, C-H stretching, C=O stretching (amide II), N-H bending (amide II), C-N stretching (amide I), C-O stretching and N-H bending, respectively [37,38].

From Figure 1D, the intensity of characteristic absorption peaks of GA and WPI changed with the different ratios of GA:WPI and the characteristic absorption peaks of CH (2927 cm^−1^, 2855 cm^−1^, 1736 cm^−1^, 1626 cm^−1^) were observed in all the microcapsules. Compared to the CH spectrum, the absorption peaks of 2927 and 1626 cm^−1^ emerged a redshift, which indicates that the vibration of phytol residues and aromatic system of CH molecule was restrained. Moreover, the absorption peak shape of hydroxyl groups (3439 cm^−1^) of CH became broader, which implies that the intramolecular hydrogen bonds could be formed between CH and the composite wall materials, especially in higher GA ratio of microcapsules. Therefore, this result can explain the reason for the increased EE of CH in microcapsules of higher GA content.

### 3.4. The Morphology of Microcapsules

**Figure 2:**
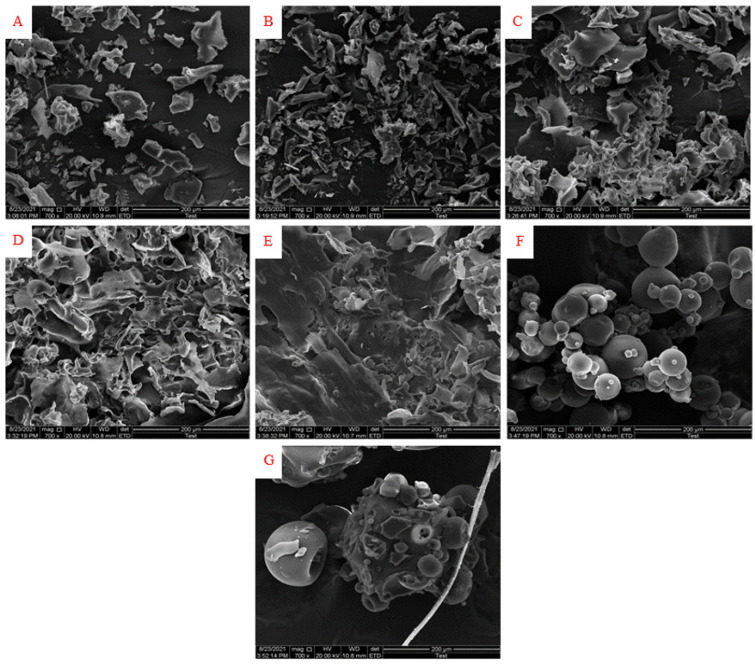
The morphology of microcapsules prepared by different ratios of GA:WPI ((**A**) GA:WPI = 1:9; (**B**) GA:WPI = 3:7; (**C**) GA:WPI = 5:5; (**D**) GA:WPI = 7:3; (**E**) GA:WPI = 9:1; (**F**) GA; (**G**) WPI).

The morphology of microcapsules prepared by different ratios of GA:WPI is shown in Figure 2. The GA and WPI powders used as controls were shaped like a wrinkled sphere. The microcapsules covered by different ratios of GA:WPI had a broken glass structure of various sizes, which was similar to previous studies where the morphology of powders was broken glassy and appeared as shriveled textures using the freeze-drying method [23,33]. Moreover, the structure of microcapsules transformed from broken glassy into irregular porous flake-like structures with the increase in GA ratios, which may be due to GA having good gelling properties, and rapid water sublimation during the freeze-drying process [39]. Moreover, the stability of the core material (CH) may be improved because the surface area was relatively reduced as the particle size increased, which could decrease the contact chance between the core material and the external environment.

### 3.5. UV Light Stability of Microcapsules

**Figure 3:**
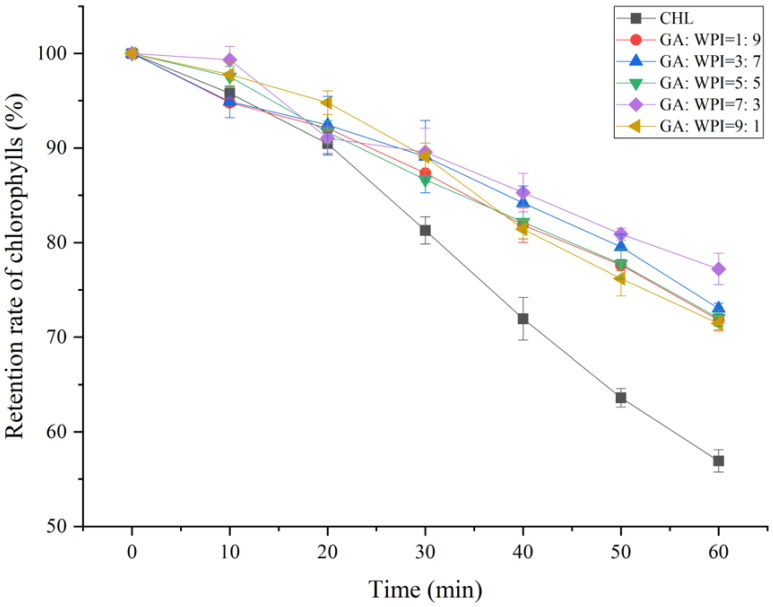
The retention rate of chlorophylls (RT_CHs_) of microcapsules covered with different ratios of GA:WPI.

The retention rate of chlorophylls (RT_CHs_) of microcapsules covered with different ratios of GA:WPI is shown in Figure 3. It is known that CH is an important photosynthetic pigment, which has the ability to transfer light energy into a chemical acceptor through changes in its molecular structure [40]. Therefore, the structural stability of CH can be evaluated by the retention rate of CH after UV irradiation. From Figure 3, the RT_CHs_ of all microcapsules covered with different ratios of GA:WPI were higher than control (without encapsulation) during the investigation period (*p* < 0.05). Moreover, the highest RT_CHs_ was observed in microcapsule with GA:WPI = 7:3 after 60 min of UV irradiation, which indicated that the CH microcapsules prepared with the formula of GA:WPI = 7:3 as composite wall material had better optical structure stability compared to other formulations. The results were consistent with previous studies that the appropriate wall material and its ratio can enhance the stability of the core material [23,41].

### 3.6. Thermal Stability of Microcapsules

**Figure 4:**
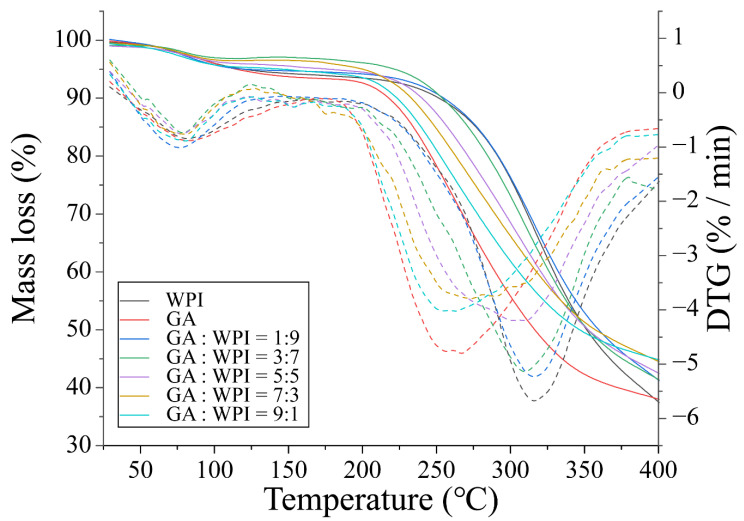
TGA and DTG patterns of GA, WPI and microcapsules are covered with different ratios of GA:WPI.

Thermogravimetric analysis (TGA) and derivative thermogravimetric analysis (DTG) patterns of GA, WPI and microcapsules covered with different ratios of GA:WPI is shown in Figure 4. All the samples showed two mass loss peaks around 73~85 °C and 260~340 °C, which may be related to adsorbed moisture in samples and the thermal degradation of each sample, respectively. Tavares et al. [42] reported that the thermal degradation above 150 to 400 °C was attributed to the decompositions and depolymerization of S-S, O-N, O-O linkages, and carbohydrate ring derived from the wall materials composite. The detailed maximum degradation temperature (T_m_) and mass loss (%) of each sample is shown in Table 4. Compared to the GA, the WPI had higher T_m_ and lower mass loss in the second thermal degradation step, which could be ascribed to the presence of more chemical bonds in the structure of WPI, such as peptide bonds and hydrophobic force [43,44]. Therefore, with the GA ratio increased, the T_m_ of the second step of microcapsules was decreased. However, the T_m_ of microcapsules at GA:WPI of 1:9 and 3:7 was higher than GA and WPI powder, which may imply that more intermolecular forces were formed between CH and the wall materials, thereby, improving the thermal stability of CH microcapsules as compared to other microcapsules. Moreover, the mass loss of peak 1 of microcapsule covered with GA:WPI of 3:7 was the lowest, and the mass loss of peak 2 was highest than that of other samples, which indicated that the intermolecular hydrogen bonds may have formed between CH and GA/WPI molecules and reduced the ability of the microcapsule to absorb of moisture molecular. This result was consistent with the previous studies that a higher GA ratio of coacervates as the wall material could have better the stability of microcapsules [42,43].

### 3.7. DPPH Free Radical Scavenging Activity of Microcapsules

**Figure 5:**
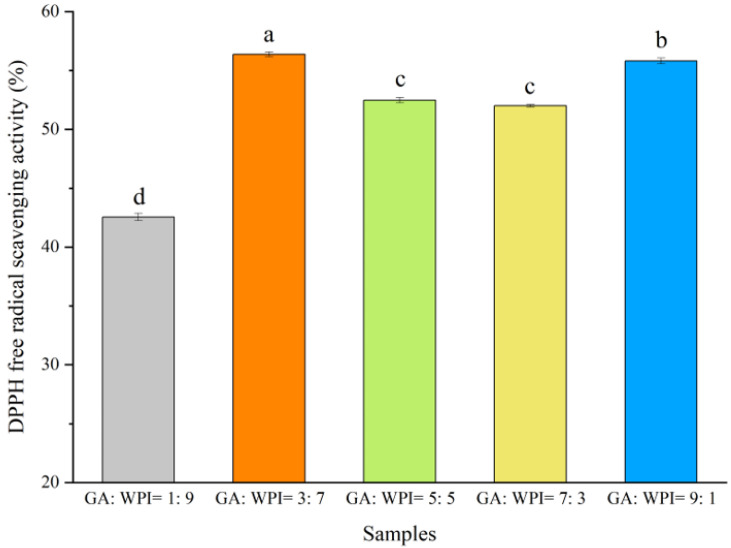
DPPH free radical scavenging activity (SA_DPPH_) of microcapsules coated with different ratios of GA:WPI (^a–d^ different letters represent the significant difference from each other (*p* < 0.05)).

DPPH free radical scavenging activity (SA_DPPH_) of microcapsules coated with different ratios of GA:WPI is shown in Figure 5. The high SA_DPPH_ of microcapsules can be associated with high CH content. From Figure 5, the microcapsules coated with GA:WPI = 3:7 showed the highest SA_DPPH_ (56.38 ± 0.19), followed by those coated with GA:WPI in ratios of 9:1, 5:5, 7:3 and 1:9, respectively (*p* < 0.05). This result can be explained based on the TGA and thermal stability of microcapsules. Kang et al. [27] reported that microcapsules with lower moisture content could inhibit the release of encapsulated materials to improve their stability. Moreover, Jamdar et al. [45] indicated that the water activity, storage temperature and light can affect the encapsulated materials content of produced microcapsules. In this study, the microcapsule coated with GA:WPI of 3:7 had the lowest moisture content and highest thermal stability, which can play an important role in the chemical stability of CH in microcapsules and can show better antioxidation activity as compared to other microcapsules coated with ratio of GA:WPI. These results were in agreement with previous studies which showed that the suitable carbohydrate and protein ratio of wall materials can present good antioxidant activity [46,47].

## 4. Conclusions

Spirulina has been a food and dietary supplement due to its many nutritional compounds and numerous health benefits. However, spirulina has undesirable odor, flavor and allergy risk, so extraction of the beneficial compounds is an effective method to expand consumption of spirulina. CHs are one of the main natural pigments of spirulina, which can be used as a potential natural additive in the food industry due to their antioxidant, anti-inflammatory, anti-bacterial and anti-carcinogenic benefits. However, CH is insoluble in water and has poorly stability, which limits its application prospects. In this study, the CH content was extracted from spirulina powder by ultrasound treatment and prepared into microcapsules with different ratios of GA:WPI (1:9, 3:7, 5:5, 7:3 and 9:1) by freeze-drying method. The effect of GA:WPI ratio on the physicochemical properties and antioxidant activity was measured to obtain better chemical stability of CH microcapsules. Results show that the color values of microcapsules prepared by GA:WPI ratio of 3:7 depicted the lowest a* negative value (−8.94 ± 0.05) and highest C* value (15.44 ± 0.08), compared with other microcapsules. Both the EE of microcapsules and the GA fraction increased. Moreover, from the absorption peaks of CH at 2927 and 1626 cm^−1^ in microcapsules emerged a redshift detected by FT-IR, which indicates that the intramolecular hydrogen bonds could be formed between CH and the composite wall materials. The SEM morphology of microcapsules showed a transition from broken fragments to irregular flake-like structures and then to structures with a porous surface as the GA ratio increased. In addition, the microcapsules coated with GA:WPI (3:7) showed the highest SA_DPPH_ (56.38 ± 0.19) due to the low moisture content and better chemical stability measured by TGA. Therefore, the GA and WPI coacervates as the wall material can improve the stability of CH extracted from spirulina, especially the GA:WPI ratio of 3:7.

## Figures and Tables

**Table 1:** The blending procedure of core matrix and wall matrix solution.

Samples	Core Matrix	Wall Matrix	
	CH Solution	GA Solution	WPI Solution
GA:WPI (1:9)	30 mL	6 mL	54 mL
GA:WPI (3:7)	30 mL	18 mL	42 mL
GA:WPI (5:5)	30 mL	30 mL	30 mL
GA:WPI (7:3)	30 mL	42 mL	18 mL
GA:WPI (9:1)	30 mL	54 mL	6 mL

**Table 2:** The visual color of microcapsules in different ratios of GA and WPI.

Sample	L*	a*	b*	C*
GA:WPI (1:9)	59.2 ± 0.53 ^b^	−8.44 ± 0.07 ^ab^	11.51 ± 0.10 ^c^	14.27 ± 0.11 ^d^
GA:WPI (3:7)	58.60 ± 0.28 ^b^	−8.94 ± 0.05 ^d^	12.59 ± 0.08 ^a^	15.44 ± 0.08 ^a^
GA:WPI (5:5)	55.32 ± 0.45 ^c^	−8.63 ± 0.08 ^c^	12.26 ± 0.11 ^b^	14.99 ± 0.12 ^b^
GA:WPI (7:3)	58.97 ± 0.71 ^b^	−8.56 ± 0.11 ^bc^	12.10 ± 0.09 ^b^	14.83 ± 0.12 ^bc^
GA:WPI (9:1)	60.89 ± 1.11 ^a^	−8.32 ± 0.07 ^a^	12.14 ± 0.16 ^b^	14.72 ± 0.15 ^c^

^a–d^ Different letters in the same column represent the significant difference from each other (*p* < 0.05).

**Table 3:** The total chlorophylls (T_CHs_), surface chlorophylls (S_CHs_) and EE of microcapsules are prepared by different ratios of GA:WPI.

Sample	T_CHS_ (μg)	S_CHs_ (μg)	EE (%)
GA:WPI (1:9)	143.67 ± 0.50 ^a^	19.38 ± 0.23 ^a^	86.51 ± 2.32 ^c^
GA:WPI (3:7)	143.67 ± 0.29 ^a^	11.17 ± 0.13 ^b^	92.22 ± 0.44 ^b^
GA:WPI (5:5)	143.58 ± 0.60 ^a^	5.72 ± 0.17 ^c^	96.02 ± 0.18 ^a^
GA:WPI (7:3)	143.69 ± 0.23 ^a^	5.69 ± 0.23 ^c^	96.04 ± 0.63 ^a^
GA:WPI (9:1)	143.58 ± 0.72 ^a^	5.56 ± 0.24 ^c^	96.13 ± 0.24 ^a^

^a–c^ Different letters in the same column represent the significant difference from each other (*p* < 0.05).

**Table 4:** The maximum weight loss temperature (T_m_) and mass loss (%) of GA, WPI and microcapsules are covered with different ratios of GA:WPI.

Sample	Peak 1		Peak 2	
	T_m_ (°C)	Mass Loss (%)	T_m_ (°C)	Mass Loss (%)
GA	79.34	6.08	262.70	55.51
WPI	83.35	5.27	315.74	56.36
GA:WPI = 1:9	73.91	5.30	332.50	52.99
GA:WPI = 3:7	77.80	2.48	312.75	55.04
GA:WPI = 5:5	78.05	3.80	305.36	52.53
GA:WPI = 7:3	75.57	3.16	282.28	51.83
GA:WPI = 9:1	77.89	4.10	263.86	50.10

## Data Availability

The data presented in this study are available within the article.

## References

[1] Marles R.J., Barrett M.L., Barnes J., Chavez M.L., Gardiner P., Ko R., Mahady G.B., Dog T.L., Sarma N.D., Giancaspro G.I. (2011). United States Pharmacopeia Safety Evaluation of Spirulina. Crit. Rev. Food Sci..

[2] Prabha S., Vijay A.K., Paul R.R., George B. (2022). Cyanobacterial biorefinery: Towards economic feasibility through the maximum valorization of biomass. Sci. Total Environ..

[3] Faieta M., Neri L., Di Michele A., Di Mattia C.D., Pittia P. (2021). High hydrostatic pressure treatment of Arthrospira (Spirulina) platensis extracts and the baroprotective effect of sugars on phycobiliproteins. Innov. Food Sci. Emerg..

[4] Fratelli C., Burck M., Assumpca Amarant M.C., Cavalcante Braga A.R. (2021). Antioxidant potential of nature’s “something blue”: Something new in the marriage of biological activity and extraction methods applied to C-phycocyanin. Trends Food Sci. Technol..

[5] Bax C.E., Chakka S., Concha J.S.S., Zeidi M., Werth V.P. (2021). The effects of immunostimulatory herbal supplements on autoimmune skin diseases. J. Am. Acad. Dermatol..

[6] Wu Q., Liu L., Miron A., Klimova B., Wan D., Kuca K. (2016). The antioxidant, immunomodulatory, and anti-inflammatory activities of Spirulina: An overview. Arch. Toxicol..

[7] Lang-Yona N., Kunert A.T., Vogel L., Kampf C.J., Bellinghausen I., Saloga J., Schink A., Ziegler K., Lucas K., Schuppan D. (2018). Fresh water, marine and terrestrial cyanobacteria display distinct allergen characteristics. Sci. Total Environ..

[8] Patel P., Jethani H., Radha C., Vijayendra S.V.N., Mudliar S.N., Sarada R., Chauhan V.S. (2019). Development of a carotenoid enriched probiotic yogurt from fresh biomass of Spirulina and its characterization. J. Food Sci. Technol..

[9] Ambati R.R., Gogisetty D., Aswathanarayana R.G., Ravi S., Bikkina P.N., Lei B., Su Y. (2019). Industrial potential of carotenoid pigments from microalgae: Current trends and future prospects. Crit. Rev. Food Sci..

[10] Soni R.A., Sudhakar K., Rana R.S. (2017). Spirulina—From growth to nutritional product: A review. Trends Food Sci. Technol..

[11] Albuquerque B.R., Oliveira M.B.P.P., Barros L., Ferreira I.C.F.R. (2021). Could fruits be a reliable source of food colorants? Pros and cons of these natural additives. Crit. Rev. Food Sci..

[12] Manivasagan P., Bharathiraja S., Moorthy M.S., Mondal S., Seo H., Lee K.D., Oh J. (2018). Marine natural pigments as potential sources for therapeutic applications. Crit. Rev. Biotechnol..

[13] Rizzi V., Gubitosa J., Fini P., Fraix A., Sortino S., Agostiano A., Cosma P. (2021). Development of Spirulina sea-weed raw extract/polyamidoamine hydrogel system as novel platform in photodynamic therapy: Photostability and photoactivity of chlorophyll a. Mater. Sci. Eng. C-Mater..

[14] Chaiareekitwat S., Latif S., Mahayothee B., Khuwijitjaru P., Nagle M., Amawan S., Mueller J. (2022). Protein composition, chlorophyll, carotenoids, and cyanide content of cassava leaves (Manihot esculenta Crantz) as influenced by cultivar, plant age, and leaf position. Food Chem..

[15] Sigurdson G.T., Tang P., Giusti M.M. (2017). Natural colorants: Food colorants from natural sources. Annu. Rev. Food Sci. Technol..

[16] Cortez R., Luna-Vital D.A., Margulis D., de Mejia E.G. (2017). Natural Pigments: Stabilization Methods of Anthocyanins for Food Applications. Compr. Rev. Food Sci. Food Saf..

[17] Santra K., Song A., Petrich J.W., Rasmussen M.A. (2021). The degradation of chlorophyll pigments in dairy silage: The timeline of anaerobic fermentation. J. Sci. Food Agric..

[18] Wang R., Ding S., Hu X., Zhang Y. (2018). Stability of chlorophyll-protein complex (photosystem II) in processed spinach: Effect of high hydrostatic pressure. Int. J. Food Prop..

[19] Ribeiro J.S., Veloso C.M. (2021). Microencapsulation of natural dyes with biopolymers for application in food: A review. Food Hydrocolloid..

[20] Saifullah M., Shishir M.R.I., Ferdowsi R., Rahman M.R.T., Quan Van V. (2019). Micro and nano encapsulation, retention and controlled release of flavor and aroma compounds: A critical review. Trends Food Sci. Technol..

[21] Rodriguez-Amaya D.B. (2019). Update on natural food pigments—A mini-review on carotenoids, anthocyanins, and betalains. Food Res. Int..

[22] Assadpour E., Jafari S.M. (2019). Advances in Spray-Drying Encapsulation of Food Bioactive Ingredients: From Microcapsules to Nanocapsules. Annu. Rev. Food Sci. Technol..

[23] Al-Maqtari Q.A., Mohammed J.K., Mahdi A.A., Al-Ansi W., Zhang M., Al-Adeeb A., Wei M., Phyo H.M., Yao W. (2021). Physicochemical properties, microstructure, and storage stability of Pulicaria jaubertii extract microencapsulated with different protein biopolymers and gum arabic as wall materials. Int. J. Biol. Macromol..

[24] Zhang Z.-H., Peng H., Woo M.W., Zeng X.-A., Brennan M., Brennan C.S. (2020). Preparation and characterization of whey protein isolate-chlorophyll microcapsules by spray drying: Effect of WPI ratios on the physicochemical and antioxidant properties. J. Food Eng..

[25] Zhang Z.-H., Peng H., Ma H., Zeng X.-A. (2019). Effect of inlet air drying temperatures on the physicochemical properties and antioxidant activity of whey protein isolate-kale leaves chlorophyll (WPI-CH) microcapsules. J. Food Eng..

[26] Wang B., Wei W., Zhang Y., Xu H., Ma H. (2022). Decontamination and quality assessment of freshly squeezed grape juice under spiral continuous flow-through pulsed light (SCFPL) treatment. J Food Process. Pres..

[27] Kang Y.-R., Lee Y.-K., Kim Y.J., Chang Y.H. (2019). Characterization and storage stability of chlorophylls microencapsulated in different combination of gum Arabic and maltodextrin. Food Chem..

[28] Bule M.V., Singhal R.S., Kennedy J.F. (2010). Microencapsulation of ubiquinone-10 in carbohydrate matrices for improved stability. Carbohyd. Polym..

[29] Chen T.T., Zhang Z.-H., Wang Z.-W., Chen Z.-L., Ma H., Yan J.-K. (2021). Effects of ultrasound modification at different frequency modes on physicochemical, structural, functional, and biological properties of citrus pectin. Food Hydrocolloid..

[30] Li X., Zhang Z.-H., Qiao J., Qu W., Wang M.-S., Gao X., Zhang C., Brennan C.S., Qi X. (2022). Improvement of betalains stability extracted from red dragon fruit peel by ultrasound-assisted microencapsulation with maltodextrin. Ultrason. Sonochem..

[31] Tavares L., Souza H.K.S., Gonçalves M.P., Rocha C.M.R. (2021). Physicochemical and microstructural properties of composite edible film obtained by complex coacervation between chitosan and whey protein isolate. Food Hydrocolloid..

[32] Erdem B.G., Kaya S. (2021). Production and application of freeze dried biocomposite coating powders from sunflower oil and soy protein or whey protein isolates. Food Chem..

[33] Pan-utai W., Iamtham S. (2020). Enhanced Microencapsulation of C-Phycocyanin from Arthrospira by Freeze-Drying with Different Wall Materials. Food Technol. Biotechnol..

[34] Cilek B., Luca A., Hasirci V., Sahin S., Sumnu G. (2012). Microencapsulation of phenolic compounds extracted from sour cherry pomace: Effect of formulation, ultrasonication time and core to coating ratio. Eur. Food Res. Technol..

[35] Chavoshizadeh S., Pirsa S., Mohtarami F. (2020). Conducting/smart color film based on wheat gluten/chlorophyll/polypyrrole nanocomposite. Food Packag. Shelf..

[36] Baiocco D., Preece J.A., Zhang Z. (2021). Encapsulation of hexylsalicylate in an animal-free chitosan-gum Arabic shell by complex coacervation. Colloid Surf. A.

[37] Han C., Xiao Y., Liu E., Su Z., Meng X., Liu B. (2020). Preparation of Ca-alginate-whey protein isolate microcapsules for protection and delivery of *L. bulgaricus* and *L. paracasei*. Int. J. Biol. Macromol..

[38] Li K., Pan B., Ma L., Miao S., Ji J. (2020). Effect of Dextrose Equivalent on Maltodextrin/Whey Protein Spray-Dried Powder Microcapsules and Dynamic Release of Loaded Flavor during Storage and Powder Rehydration. Foods.

[39] Parthasarathi S., Anandharamakrishnan C. (2016). Enhancement of oral bioavailability of vitamin E by spray-freeze drying of whey protein microcapsules. Food Bioprod. Process..

[40] Chen M., Blankenship R.E. (2011). Expanding the solar spectrum used by photosynthesis. Trends Plant Sci..

[41] Gallardo G., Guida L., Martinez V., López M.C., Bernhardt D., Blasco R., Pedroza-Islas R., Hermida L.G. (2013). Microencapsulation of linseed oil by spray drying for functional food application. Food Res. Int..

[42] Tavares L., Barbosa Barros H.L., Pacheco Vaghetti J.C., Zapata Norena C.P. (2019). Microencapsulation of Garlic Extract by Complex Coacervation Using Whey Protein Isolate/Chitosan and Gum Arabic/Chitosan as Wall Materials: Influence of Anionic Biopolymers on the Physicochemical and Structural Properties of Microparticles. Food Bioprocess Technol..

[43] Tavares L., Norena C.P.Z. (2020). Encapsulation of Ginger Essential Oil Using Complex Coacervation Method: Coacervate Formation, Rheological Property, and Physicochemical Characterization. Food Bioprocess Technol..

[44] Falsafi S.R., Rostamabadi H., Nishinari K., Amani R., Jafari S.M. (2022). The role of emulsification strategy on the electrospinning of beta-carotene-loaded emulsions stabilized by gum Arabic and whey protein isolate. Food Chem..

[45] Jamdar F., Ali Mortazavi S., Reza Saiedi Asl M., Sharifi A. (2021). Physicochemical properties and enzymatic activity of wheat germ extract microencapsulated with spray and freeze drying. Food Sci. Nutr..

[46] Mansour M., Salah M., Xu X. (2020). Effect of microencapsulation using soy protein isolate and gum arabic as wall material on red raspberry anthocyanin stability, characterization, and simulated gastrointestinal conditions. Ultrason. Sonochem..

[47] Elsebaie E.M., Essa R.Y. (2018). Microencapsulation of red onion peel polyphenols fractions by freeze drying technicality and its application in cake. J. Food Process. Preserv..

